# Identification of the Anti-Aflatoxinogenic Activity of *Micromeria graeca* and Elucidation of Its Molecular Mechanism in *Aspergillus flavus*

**DOI:** 10.3390/toxins9030087

**Published:** 2017-03-01

**Authors:** Rhoda El Khoury, Isaura Caceres, Olivier Puel, Sylviane Bailly, Ali Atoui, Isabelle P. Oswald, André El Khoury, Jean-Denis Bailly

**Affiliations:** 1 Toxalim, Université de Toulouse, INRA, ENVT, INP Purpan, UPS, Toulouse F-31027, France; rhodakhoury@gmail.com (R.E.K.); isauracaceres@hotmail.com (I.C.); olivier.puel@inra.fr (O.P.); s.bailly@envt.fr (S.B.); isabelle.oswald@inra.fr (I.P.O.); 2 Laboratoire de Mycologie et Sécurité des Aliments (LMSA), Département des sciences de la vie et de la terres - Biochimie, Faculté des Sciences, Université Saint-Joseph, P.O. Box 17-5208, Mar Mikhael Beirut 1104 2020 Lebanon; andre.khoury@usj.edu.lb; 3 Laboratory of Microbiology, Department of Natural Sciences and Earth, Faculty of Sciences I, Lebanese University, Hadath Campus, P.O. Box 5, Beirut, Lebanon; a.atoui@cnrs.edu.lb

**Keywords:** Aflatoxin B_1_, *Aspergillus flavus*, hyssop, inhibition, oxidative stress

## Abstract

Of all the food-contaminating mycotoxins, aflatoxins, and most notably aflatoxin B_1_ (AFB_1_), are found to be the most toxic and economically costly. Green farming is striving to replace fungicides and develop natural preventive strategies to minimize crop contamination by these toxic fungal metabolites. In this study, we demonstrated that an aqueous extract of the medicinal plant *Micromeria graeca*—known as hyssop—completely inhibits aflatoxin production by *Aspergillus flavus* without reducing fungal growth. The molecular inhibitory mechanism was explored by analyzing the expression of 61 genes, including 27 aflatoxin biosynthesis cluster genes and 34 secondary metabolism regulatory genes. This analysis revealed a three-fold down-regulation of *aflR* and *aflS* encoding the two internal cluster co-activators, resulting in a drastic repression of all aflatoxin biosynthesis genes. Hyssop also targeted fifteen regulatory genes, including *veA* and *mtfA*, two major global-regulating transcription factors. The effect of this extract is also linked to a transcriptomic variation of several genes required for the response to oxidative stress such as *msnA*, *srrA*, *catA*, *cat2*, *sod1*, *mnsod*, and *stuA*. In conclusion, hyssop inhibits AFB_1_ synthesis at the transcriptomic level. This aqueous extract is a promising natural-based solution to control AFB_1_ contamination.

## 1. Introduction

*Aspergillus flavus*, a saprophytic fungus that develops on many crops including maize, oilseed, dried fruit, and spices [1], is the main producer of aflatoxin B_1_ (AFB_1_), the most potent naturally occurring carcinogen. AFB_1_ is associated with several pathologies mainly targeting the liver [2]. This mycotoxin also has major economic impacts as it contributes to considerable amounts of crop and livestock losses occur [3], endangering food and feed security. Globalization of food trade and global climate changes have further exacerbated the situation [4,5].

Many methodologies have been developed to limit AFB_1_ contamination in crops. First, the implementation of good agricultural practices is undoubtedly a key factor to reduce undesirable growth of fungi. Fungal growth and mycotoxin production closely depend on temperature and humidity [6] and since these meteorological parameters are impossible to control, contamination cannot be completely avoided. The massive use of fungicides in crops over the last decades led to an accumulation of toxic chemical residues in food products but also in water and soil and resulted in the development of resistant pathogen populations [7]. 

Recent studies have pointed out the use of physio-chemical approaches to counteract aflatoxin contamination [8,9] while others were based on the detoxification properties or the protective physiological effect of bacterial metabolites or natural extracts used as feed supplements [10,11,12,13]. Although proven to be efficient, these approaches remain strictly restricted to animal feed. Nonetheless, such alternative strategies could be used at a much earlier phase, postharvest, to prevent aflatoxin production in crops. As an example, several bio-control approaches were developed relying on the use of microorganisms, such as lactic acid bacteria strains [14] or atoxinogenic fungi [15,16]. These strains displayed the ability to inhibit aflatoxin production or fungal growth to a certain extent [17,18]. Prevention strategies could also rely on the use of natural substances like plant extracts or essential oils. As plants grow, they produce many metabolites that serve as a defense against a number of environmental stresses. Therefore, plant extracts have long been studied as protective bioactive agents and it was demonstrated that some of them have antifungal or anti-toxinogenic properties [19,20]. 

*Micromeria graeca*, commonly known as hyssop, is an herbaceous plant belonging to the *Lamiaceae* family. It is a widespread species in the Mediterranean basin and is frequently used for medicinal purposes and as a condiment [21]. The composition of essential oil, the toxicity, and the antimicrobial effect of different species of hyssop were previously described [22,23,24,25,26,27], but there is little data on the composition and toxicity of the aqueous extract of hyssop [21,28], and none on its antifungal or anti-mycotoxinogenic effect. The purpose of this study was to test the aqueous extract of *M. graeca* for its ability to prevent aflatoxin’s biosynthesis. We observed that it inhibits AFB_1_’s production by *A. flavus* strains without interfering with fungal growth. The molecular mechanism of action of this extract involved down-regulation of all the AFB_1_ biosynthetic cluster as well as modulation in the expression of 15 secondary metabolite regulating genes.

## 2. Results

### 2.1. Effect of Aqueous Extract of Hyssop on the Production of AFB_1_ and the Development of A. flavus 

**Figure 1:**
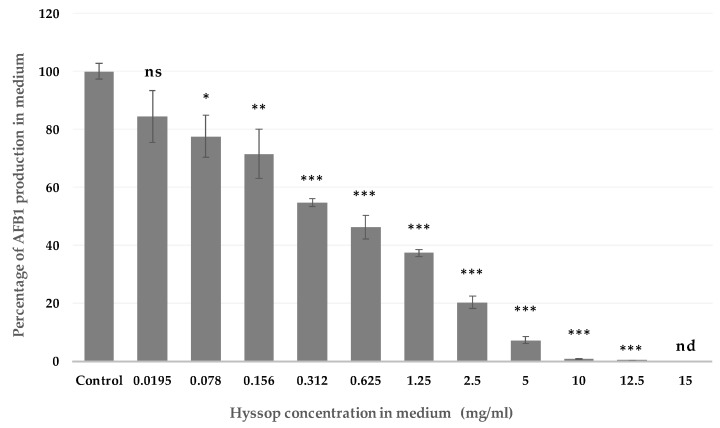
Aflatoxin B_1_ (AFB_1_) production as a function of hyssop concentration. Malt extract agar (MEA) medium was supplemented with increasing concentrations of hyssop extract ranging from 0.0195 to 15 mg/mL and cultivated at 27 °C, in the dark, for 8 days. AFB_1_ concentrations were quantified through HPLC/FLD. Results are expressed as mean % ± SEM (*n* = 3). ns = no significant changes; nd = not detectable; * *p*-value < 0.05; ** *p*-value < 0.01; *** *p*-value < 0.001.

When *A. flavus* strain NRRL 62477 was grown in a hyssop-supplemented medium, a dose-dependent decrease in AFB_1_ production was observed. The downward trend started at the lowest concentration (0.0195 mg/mL) and was statistically significant (28.5%, *p*-value 0.0032) starting at 0.078 mg/mL. Inhibition reached 99.2% at 10 mg/mL and AFB_1_ was no longer detectable at 15 mg/mL (Figure 1). Further experiments were then conducted supplementing the culture medium with 10 mg/mL of hyssop extract, this being the lowest to present a quasi-total inhibition of AFB_1_ synthesis. 

At 10 mg/mL, aflatoxin inhibition by hyssop was accompanied by a mild increase of the colony diameter (4.4 ± 0.03 vs. 4.25 ± 0.03 cm for treated and control respectively, *p*-value 0.0213). However, no significant increase in the total spore count or in the spore density was observed following the addition of hyssop. Besides, no further change was observed in mycelium weights or for the germination delay in the presence of hyssop in the medium (Table 1).

**Figure 2:**
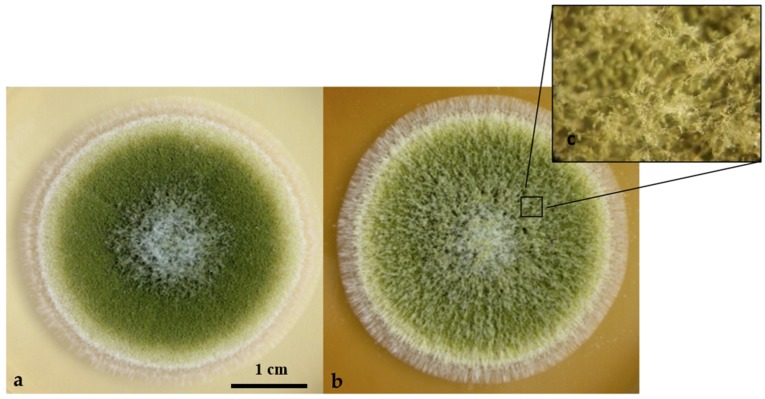
Phenotype of *A. flavus* strain NRRL62477 after four days of culture at 27 °C in MEA medium or MEA medium supplemented with 10 mg/mL of hyssop aqueous solution. (**a**) Control culture grown on a regular MEA medium; (**b**) MEA medium was supplemented with 10 mg/mL of aqueous solution of hyssop; (**c**) Magnification of the aerial mycelium covering the hyssop treated culture.

Following addition of hyssop in medium, *A. flavus* colonies presented numerous macro and microscopic modifications. The major noticeable macroscopic morphological change was the development of an abundant aerial mycelium covering the entire surface of the colony. This latter also displayed fasciculation on the edge. The presence of these numerous floccose tufts also increased the depth of the colony (Figure 2).

**Figure 3:**
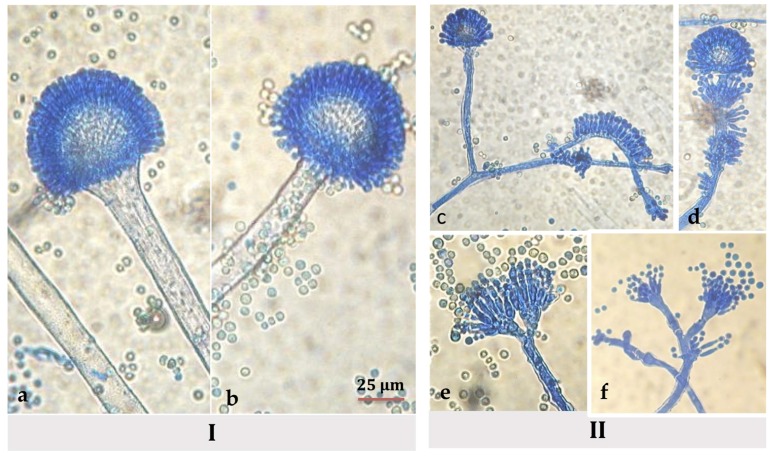
Microscopic views (x400) of *A. flavus* NRRL 62477 conidiophores in the (I) basal mycelium on (**a**) MEA medium and (**b**) MEA supplemented with 10 mg/mL of hyssop extract, and the (II) aerial mycelium showing the development of anarchic philalides when strain was grown on a hyssop-treated MEA medium; (**c**) and (**d**) development of anarchic philalides; (**e**) conidiophore bearing two vesicles and (**f**) presence of short conidiophores with columnar heads.

Under microscope, classic *A. flavus* structures were present in the basal mycelium of hyssop-treated cultures: long, coarse, un-branched conidiophores and radiate biseriate conidial heads. However, in the aerial mycelium, conidiophores, vesicles, and conidia presented an atypical morphology and organization: (1) an increased number of short conidiophores bearing small columnar heads in relation with the abundant aerial mycelium; (2) phialides developing anarchically on hyphae and on conidiophores in the absence of a vesicle; (3) and the presence of conidiophores with two, and less frequently three, sporulated vesicles (Figure 3).

At the dose of 10 mg/mL, we observed a 77.7% and 70.8% inhibition of the production of AFB_1_ in two other *A. flavus* strains (E28 and E71respectively), without alteration of fungal growth. Similar morphological changes were also observed for these two strains (data not shown). 

### 2.2. Aqueous Extract of Hyssop Down-Regulated the Expression of AFB_1_ Cluster Genes

**Figure 4:**
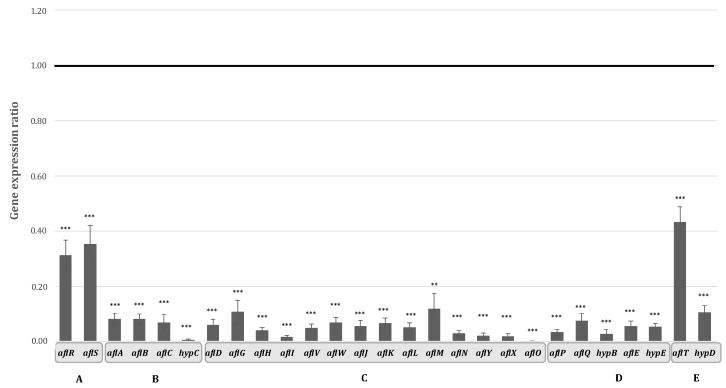
Expression of genes belonging to AFB_1_ cluster genes in the presence of 10 mg/mL of hyssop aqueous extract; (**A**) internal cluster regulators (**B**) genes involved in the earlier steps of AFB_1_ enzymatic cascade leading to the formation of norsolorinic acid (**C**) genes involved in the middle steps of the AFB_1_ enzymatic cascade converting norsolorinic acid into sterigmatocystin (**D**) genes involved in the final steps of the cascade leading to AFB_1_ synthesis (**E**) genes with uncharacterized functions. Black line represents the expression level of control. ** *p*-value < 0.01; *** *p*-value < 0.001.

The biosynthesis of AFB_1_ is the result of a well-described cascade of more than 20 enzymatic reactions. This cascade is governed by 27 clustered genes encoding the corresponding enzymes as well as two internal regulators, AflR encoding a Gal4 zinc finger transcription factor and its co-activator AflS. The expression of all of these genes was analyzed in order to determine whether the inhibition of AFB_1_ synthesis occurred at a transcriptomic level. Inhibition of AFB_1_ production in hyssop-supplemented media was accompanied by a decrease in the expression of both of *aflR* and *aflS* genes by 3.2 and 2.8 times respectively (*p*-value < 0.0001). Apart from *aflT* (encoding a MFS-family transporter), which is not regulated by the AflR/AflS complex [29] and was down regulated only by 2.3 times (*p*-value < 0.0001), the expression of the remaining cluster genes was severely repressed (Figure 4).

Genes undergoing the most drastic inhibitions were *hypC*, *aflI*, and *aflO*, encoding enzymes respectively intervening at the beginning, middle, and end of the biosynthetic pathway and with corresponding fold-changes of 167.2, 60.7, and 468.8 with *p*-values < 0.001. For the genes encoding enzymes involved in the first steps of the cascade leading to the polyketide structure, *aflA*, *aflB*, and *aflC*, expression was decreased by 12.2, 12.3, and 14.7 respectively. The least impacted genes were *aflM*, *aflG*, and *hypD* with expression levels decreased by 8.4, 9.3, and 9.4 times respectively. For the remaining AFB_1_ cluster genes, the same downward trend was observed with expression levels decreased by 14 to 50 folds (Supplementary Material Table S1). This repression of the entire aflatoxin gene cluster could then be directly linked to the inhibition of the production of this mycotoxin. 

### 2.3. Transcriptomic Effect of Hyssop Extract on the Expression of Genes Coding for Regulators of Secondary Metabolites

**Figure 5:**
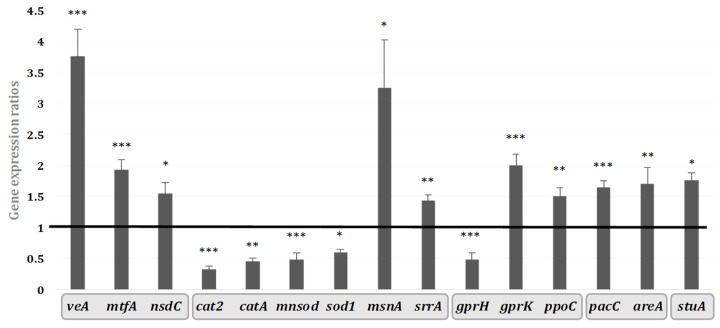
Schematic representation of gene expression ratios of the different regulatory genes affected upon 10 mg/mL hyssop supplementation of MEA media. Genes are grouped into the five categories described above. The black line represents the expression level of genes in control cultures. * *p*-value < 0.05; ** *p*-value < 0.01; *** *p*-value < 0.001.

The expression of AFB_1_ cluster genes is subjected to the control exerted by regulatory factors encoded by genes outside of the cluster. In order to investigate a possible genetic relationship between those regulators and the transcriptional inhibition of AFB_1_ cluster genes after the addition of hyssop extract, we conducted a study on the regulatory network affecting secondary metabolism that includes 34 genes involved in several fungal functional pathways. Among these, a total of 15 genes involved in diverse cellular mechanisms were modulated by adding hyssop to the culture medium (Figure 5). The modulated genes could be grouped in five categories based on their cellular functions in other ascomycetes [30]. The first one includes global regulating factors such as *veA*, *mtfA*, *nsdC* that were affected with expression levels respectively increased by 3.8, 1.9, and 1.5 folds (*p*-values < 0.0001, 0.0001 and 0.0122). Secondly, genes encoding enzymes involved in cellular protection from oxidative stress such as superoxide dismutases (*sod1* and *mnsod*) and catalases (*catA* and *cat2*) had their expression decreased by 1.6, 2, 2.2, and 3 folds respectively (*p*-values 0.013, 0.0007, 0.004, and < 0.0001). Other genes intervening in the oxidative stress response and encoding transcription factors, notably *msnA* and *srrA*, had their expression levels increased by 3.2 and 1.4 times with *p*-values of 0.0126 and 0.0017, respectively. The third category involves *gprK* and *gprH*, encoding G-protein receptors involved in relaying external signals with *gprK* expression increased by 2 folds (*p*-value < 0.0001) and *gprH* decreased by 2.1 folds (*p*-value 0.0006). Moreover, *ppoC* encoding a fatty acid dioxygenase involved in oxylipin production also presented an expression decreasing by 1.5 times (*p*-value 0.003). The conidial developmental factor *stuA* whose expression increased by 1.8 times (*p*-value 0.0012) constitutes the fourth category. Finally, two genes encoding environmentally influenced transcription factors whose expression is respectively modulated by nitrogen and medium pH, *areA* and *pacC* were also triggered by the addition of hyssop in the medium and their expression levels were respectively increased by 1.7 and 1.6 folds with *p*-values of 0.0215 and < 0.0001. 

## 3. Discussion

### 3.1. Hyssop Leads to an Inhibition of AFB_1_ Synthesis in A. flavus by a Transcriptomic Regulation of AFB_1_ Cluster Genes

The inhibition of AFB_1_ synthesis was dose-dependent (Figure 1) and the same impact was observed using several strains of *A. flavus*. Another recent study by Omidpanah et al. (2015) [31] pointed out that some aqueous extracts including thyme and mint had fungicidal effect on *A. flavus* at concentrations of 0.2 and 0.8 mg/mL respectively, yet without determining aflatoxin inhibition at any of the concentration range used. At a comparable concentration of 0.625 mg/mL, hyssop’s extract was able to inhibit the production of aflatoxin by 52% without restraining the growth of *A. flavus*. A slight trend for an increase in total spore count and density was observed upon addition of hyssop, but these results remain statistically non-significant and do not affect the anti-aflatoxinogenic property of the extract. 

AFB_1_ biosynthesis in aflatoxinogenic fungi is the result of a coordinated cascade of enzymatic reactions. The enzymes catalyzing these reactions are encoded by 27 genes and grouped into a cluster located in the telomeric region of the 3rd chromosome of aflatoxinogenic species [32]. The function of almost all of these genes was elucidated and described in previous works [33,34,35]. We demonstrated that AFB_1_ inhibition in *A. flavus* by *M. graeca* extract is consistent with a drastic repression of all AFB_1_ cluster genes following a significant decrease in the expression levels of the two internal cluster regulators *aflR* and *aflS* (previously annotated as *aflJ*) (Figure 4). Furthermore, the down-regulation of these two latter genes in AF-repressive conditions has been previously described as associated with the repression of the entire AF cluster genes [36]. It could also be noted that the inhibition level of the cluster genes does not depend on their chronological intervention in the enzymatic cascade leading to AFB_1_ synthesis, contrary to what has been described for other anti-aflatoxinogenic agents such as eugenol [30].

### 3.2. The Implication of VeA and MtfA: Two Leading Transcriptional Regulators

VeA is a global regulating transcription factor involved in primary and secondary metabolism [37] and recruiting other factors such as LaeA and VelB to form the trimeric velvet complex. The activity of this complex affects fungal development, conidiation, and secondary metabolism [38]. In hyssop-treated cultures, transcripts of *laeA*, *velB*, and *vosA*, the latter being an interacting partner of *velB* [39], were not affected (Table S1). This result further highlights the independent role of VeA in multiple other cellular mechanisms [40]. The presence of VeA is necessary for the expression of secondary metabolite genes; however, it can also act as a repressor of some of these genes and thus inhibit the production of the concerned metabolite. VeA is essential for the transcription of AF cluster genes, including the transcription factor *aflR* and others (*aflD*, *aflM*, and *aflP*) regulating production of aflatoxin. Deletion of the *veA* gene led to the repression of AFB_1_ cluster genes in *A. flavus* [41]. However, according to our current study and to another recent one [30], a repression of all AFB_1_ cluster genes can also coincide with a *veA*-over-expression profile (Figure 5). Such is the case of penicillin, produced by *A. nidulans* where an OE:*veA* led to the repression of *acvA*, the penicillin biosynthesis gene and subsequent inhibition of penicillin production [42].

VeA can also interact with another conserved global transcription factor, MtfA. The latter has a major role in regulating the development and the secondary metabolism in filamentous fungi [43] and it is linked to AFB_1_’s biosynthesis as well as to *aflR*’s expression. An over-expression of *mtfA* in *A. flavus* has drastically inhibited the production of AFB_1_ following a down-regulation of *aflR*, whereas the effect of an *mtfA* deletion was less important [44]. The concurring over-expression of *mtfA* and *veA* in our conditions (Figure 5), which further highlights the interaction between these two factors, could then be responsible for down-regulating AF-cluster genes and inhibiting the production of aflatoxin. 

### 3.3. Implication of Other Regulatory Factors in AF Inhibition by M. graeca—Hyssop Extract 

Stressful environmental conditions may lead fungi to establish several defense lines to limit cellular damage. For example, when subjected to oxidative stress, fungi might respond by producing secondary metabolites such as aflatoxins. The oxidative stress generated by enhanced lipid peroxidation and the free radical generation was proven to be a prerequisite for aflatoxin production in *A. parasiticus* [45]. Moreover, the anti-aflatoxinogenic effect of antioxidant agents, such as eugenol, seems to be linked to an alleviation of oxidative stress and lipid peroxidation as well as a modulation of the expression of a number of genes involved in the oxidative stress response [30,46]. 

It has been demonstrated that VeA contributes to a positive transcriptomic modulation of stress-tolerance genes such as *msnA* and *srrA* under induced oxidative stress conditions [39]. The expression of these two transcription factors is then highly dependent on that of *veA.* Therefore, their over-expression in a hyssop-treated medium might be the outcome of an over-expressed *veA* (Figure 5). The developmental factor StuA has also been associated to stress-response in fungi, yet there isn’t a clear view on its contribution [47]. However, its dependence on *msnA* was shown since its expression levels were modulated in *A. flavus* as well as in *A. parasiticus msnA*-deleted strains [48]. MsnA is also known for regulating the expression of the catalases (CAT)- and superoxide dismutases (SOD)-encoding genes [48]. Those antioxidant enzymes along with aflatoxin formation are suggested as part of the fungus defense mechanism against reactive oxygen species (ROS) damages [49]. When the medium was supplemented with *M. graeca* extract, *A. flavus* responded by decreasing the expression of SOD- and CAT-encoding genes such as *sod1*, *mnsod*, *catA* and *cat2* as levels of *msnA* increased thus resulting in an AF-biosynthesis repression, possibly related to an alleviation of environmental oxidative stress. 

Two other regulatory factors that were not linked to oxidative stress were also modulated by hyssop’s addition to medium. The first regulator is PacC, which is a pH-dependent factor whose over-expression in aflatoxin-repressive conditions is to be investigated. In fact, PacC is usually activated under alkaline conditions [50] whereas hyssop addition does not change the pH of the medium (data not shown).

The second regulatory factor is NsdC, known to be a developmental regulator whose alteration causes several morphological aberrances such as shorter-stipe conidiophores presenting abnormal conidial-head formations. Similar to VeA, NsdC modulation could participate to the morphological modifications observed in hyssop-treated cultures (Figure 3). It has also been linked to aflatoxin cluster-gene expression [51] as well as being a global regulator of secondary metabolism [52]. More data is yet to be collected on the individual and possibly collaborative roles of both of these factors in the regulation of secondary metabolism.

### 3.4. Morphological Modifications of Conidiophores and Vesicles of A. flavus in Hyssop-Supplemented Media

Besides its role in secondary metabolite regulation, VeA is also a developmental factor in regulating morphogenesis as alterations in its expression levels can result in morphological abnormalities. For example, a reduction in fungal aerial hyphae was noted in both *A. flavus* and *Fusarium graminaerum veA* deleted strains [41,53]. Therefore, the modulation of *veA* expression could contribute to morphological abnormalities observed upon hyssop exposure (Figure 3).

Furthermore, a single previous study has described the modification of the aerial hyphae in an AF-inhibiting profile in *A. parasiticus* in the presence of n-decyl aldehyde, a corn-derived volatile compound [54]. However, another study conducted on *A. flavus* mutant strains described the appearance of morphological abnormalities, notably on phialide formation, associated to a cessation of AFB_1_ production [51].

Alterations in morphology, such as the development of aerial hyphae, were also associated to an imbalance in the G-protein signal transduction pathway [55,56]. This pathway is governed by the binding of signaling molecule to G-protein coupled receptors (GPCR) such as those encoded by *gprK* and *gprH*, both affected by the addition of hyssop to the media (Figure 5), and tuned by regulators of the G-protein signaling cascade (RGS), whose roles and implication in AFB_1_ synthesis are being investigated in *A. flavus* [57]. G-protein signaling pathway is also linked to oxylipins that are hormonal-like signaling molecules [58] produced by fatty-acid-oxygenases such as PpoC. Moreover, oxylipins regulation has also been described as VeA-dependent [37]. However, fungal signal perception and transduction pathways are a very complex loop due to the diversity of signals that might initiate them and most importantly to the numerous acting factors involved downstream. Since *M. graeca* extract is a complex extract containing many signal-provoking agents such as polysaccharides, amino acids, minerals, phenolic compounds and many others, it remains possible that morphological modifications have no direct link to AFB_1_’s inhibition.

## 4. Conclusions

This study demonstrates the efficiency of *M. graeca* aqueous extract in limiting AFB_1_ contamination without altering fungal growth. Such an effect could ensure food safety without affecting biodiversity. Indeed, *A. flavus* is a very competitive crop-contaminating agent; therefore, the use of fungistatic agents could favor the emergence of other, possibly uncontrollable microorganisms. According to our results, inhibition by hyssop extract occurs at a transcriptomic level as expression ratios of all of aflatoxin cluster-genes were severely decreased. Nonetheless, hyssop extract triggered a response in several fungal cellular mechanisms including cellular signaling, global transcription factors, and conidial development, as well as factors acting in the oxidative stress response. Massive transcriptomic analyses, such as RNAseq or Microarray assays, would be a good complement to this study since they could provide a broader vision of cellular functions affected by the addition of hyssop to the medium. Nonetheless, being as complex as it is, this extract may shelter several bioactive compounds [59] contributing in a complementary way to its anti-aflatoxinogenic activity. To ascertain more accurately the inhibitory mechanism of action, the content of this extract needs to be deciphered in order to determine and purify its active molecules as well as the inhibition extent of each of the isolated compounds. 

## 5. Materials and Methods 

### 5.1. Solvents and Standards 

All solvents were HPLC grade and purchased from ThermoScientific Fisher (Villebon-Sur-Yvette, France). Lyophilized aflatoxin B_1_ standard was purchased from Sigma Aldrich (St. Louis, MO, USA). Stock solutions of each of the standards were prepared in methanol and stored at 4 °C in the dark. Calibration curves were prepared beforehand by diluting stock solutions with mobile phase used for HPLC analysis.

### 5.2. Preparation of the Aqueous Solution of Hyssop 

Dried hyssop (*M. graeca)* was commercially purchased from Tyr, Lebanon. Hyssop species was kindly confirmed by Prof. Marc Beyrouthy (Department of Agricultural Sciences, USEK—Lebanon). Leaves were ground with an electrical grinder and ten grams of ground hyssop were added to 80 mL of bi-distilled water and placed on a horizontal shaking table at 220 rpm for 24 h. Extracts were then filtered through cotton gauze before being centrifuged for 10 min at 3500 rpm. Filtrates were centrifuged once again, at 4700 rpm for 30 min and autoclaved at 121 °C for 15 min. Final sterile extracts were stored at +4 °C until their use. 

### 5.3. Fungal Strains and Growth Conditions 

A referenced toxinogenic *Aspergillus flavus* strain NRRL 62477 isolated from paprika samples harvested from a Moroccan market [59] was used to evaluate aflatoxin inhibition by aqueous solution of hyssop as well as the molecular mechanism of inhibition. Further analysis of total aflatoxin inhibition by the aqueous hyssop solution was conducted on two other *A. flavus* strains (E28 and E71) that were previously isolated from white pepper and paprika samples from Morocco [60]. Strains were cultivated on a malt extract agar (MEA) medium (30 g malt extract and 15 g agar-agar per liter) (Biokar Diagnostics, Allone, France), supplemented at 2% *v*/*v* with the autoclaved aqueous hyssop solution, whereas 2% *v*/*v* water-supplemented media were used for control cultures. The media for RNA isolation and dry weight measurement were layered with 8.5 cm diameter cellophane disks (Hutchinson, Chalette-sur-Loing, France) before inoculation in order to allow separation of mycelium from the culture medium. Spore suspensions were prepared in Tween 80 (0.05% in water) from a one-week-old MEA culture. Spores were counted on a Malassez cell and 10^3^ spores were inoculated in the center of the medium. Cultures destined for RNA isolation were incubated in 6 replicates per condition for 4 days. For AFB_1_ quantification assays, cultures were incubated for 8 days and were in triplicates. All cultures were incubated at 27 °C. The media pH was measured before and after inoculation and after incubation using a H199161 food pH-meter (Hanna Instruments, Tanneries, France).

### 5.4. Examination of Cultural Parameters

#### 5.4.1. Effect on Growth

The final growth mean was estimated by the measurement of culture diameters in length and width at day 4.

#### 5.4.2. Mycelium Dry Weight 

Following a four-day incubation period, cellophane disks were peeled off and placed in new petri dishes that were incubated for 48 h at 60 °C. Dried mycelium films were allowed to cool in a desiccator before being weighed on an analytical balance. Final weight was calculated by subtracting the mean weight of four desiccated control cellophane disks.

#### 5.4.3. Total Spore Quantification 

Colonies were cut out of MEA media, 1 mm beyond the mycelium border, placed in a stomacher bag with 50 mL of Tween 0.05% and spores were gently manually scraped off of culture without tearing the media. The bag was then placed in a stomacher for 90 s. The supernatant was filtered through cotton gauze that was then rinsed with 3 × 20 mL Tween 0.05%. Spore solutions were homogenized by thorough vortex and subsequent dilutions were prepared in Tween 0.05% for counting on a Malassez cell in order to determine the total spore count (SC). Spore density (SD) was calculated as *SD* = *SC*/($\pi r^{2}$), *r* = average colony radius. 

#### 5.4.4. Delay to Germination 

Two hundred spores were inoculated in the center of the media and germinating spores were counted after a 16-h incubation period at 27 °C by stereo-microscopic examination. 

#### 5.4.5. Fungal Morphological Features 

Macroscopic (e.g., color of conidial areas, thallus margin and texture, aspect of conidial heads and colony reverse) and microscopic (e.g., conidiophore, shape of vesicles, number of sterigmata, shape of conidia and ornamentation) characters were observed under stereomicroscope SZX9—X12-120 (Olympus, Rungis, France) and optical microscope CX41—X400 and X1000 (Olympus, Rungis, France) respectively. 

### 5.5. RNA Isolation and Reverse Transcription 

Cellophane disks along with the four-day mycelium were peeled off from the medium, finely grinded with liquid nitrogen and a maximum of 100 mg were used for total RNA purification through a RNeasy Plus Minikit (Qiagen, Hilden, Germany), which includes an on-column genomic DNA clean-up, following the manufacturer’s instructions. RNA integrity and purity were checked with agarose gel electrophoresis and a NanoDrop ND1000 (Labtech, Palaiseau, France) that also determined its concentration. First-strand cDNA synthesis reaction was primed using RevertAid Reverse Transcriptase (MBI Fermentas, UK), RNase Inhibitor (Applied Biosystems, Warrington, UK) and an anchored oligo(dT) Bys 3′ Primer (5′-GCTGTCAACGATACGCTAACGTAACGGCATGACAGTGTTTTTTTTTTTTTTTTT-3′). An RT minus sample, where no reverse transcriptase reaction takes place, and a sterile water sample were added as negative controls in order to verify the absence of undesirable genomic DNA contamination and primer complementation, respectively. 

### 5.6. Real-Time PCR Expression Profile Analysis of Genes Regulating AFB_1_ Biosynthesis in A. flavus 

The genome of *A. flavus* strain NRRL 3357 (GenBank accession number EQ963478) served as a matrix for all of the primer used in this study. Gene selection and the corresponding primer pair sequences were adapted from a previous work [29] and primer sequences of the *stuA* gene (AFLA_046990) were added in this study (stuA_F: GATAAACGGAACCAAACTGCTCAA; stuA_R: CACGCTCAAATGGGATCCAA ). In total, the expression of 61 genes was simultaneously analyzed, 27 of which corresponded to the AFB_1_ cluster and 34 to regulatory factors. Regulatory factors were grouped into 6 categories: (1) environmental transcription factors (Area, CreA, MeaB and PacC); (2) oxidative stress response factors (AtfA, AP-1, CatA, Cat2, MnSOD, MsnA, and SrrA); (3) the velvet complex (LaeA, VeA, VelB and VosA); (4) factors belonging to different cellular signaling families such as the Ras-family (RasA), the G-protein signaling family (FadA and FlbA) as well as the G-protein receptors (GprK, GprA, GprH, GprP, and GprG) and oxylipin enzymes (PpoA, PpoB, and PpoC); (5) regulators of development and conidiation (FluG, BrlA, StuA, and AbaA) and (6) global secondary-metabolism-regulating transcription factors (NsdC, MtfA, and Fcr3). Primer pairs design was based on the coding sequence of the corresponding genes, with at least one of the primers extending on an exon/exon junction in order to avoid undesirable genomic DNA amplification. Primer-dimers or self-complementarities were evaluated using the PrimerExpress 2.0 software (Applied Biosystems, Courtaboeuf, France). All primers were synthesized by Sigma Aldrich (Saint-Quentin Fallavier, France). Following RNA extractions and reverse transcriptase reactions, real-time PCR assays were performed on 15 ng cDNA in a 5 μL reaction volume per well, using Power SYBR^®^ Green PCR Master Mix (Applied Biosystems, Warrington, UK) as a fluorescent dye for cDNA quantification. Master mixes and diluted cDNA samples were prepared separately on 96-well Framestar Jupe plates (Dominique Dutscher, Issy-les-Moulineaux, France) and mixed in 384-well plates by an Agilent Bravo Automated Liquid Handling Platform (Agilent Technologies, Santa Clara, CA, USA). All real time amplification reactions were carried out on a ViiA7 Real-Time PCR System (Applied Biosystems, Warrington, UK), as described by Tannous et al. [61]. 

### 5.7. Aflatoxin Extraction and HPLC Quantification 

Media of four- and eight-day cultures were entirely retrieved and their AFB_1_ content determined after extraction with 25 and 40 mL chloroform respectively. Extracts were held for 2 h on a horizontal shaking table at 200 rpm and were then filtered through a Whatman 1PS phase separator filter (GE Healthcare Life Sciences, Vélizy-Villacoublay, France, 150 mm diameter). Filtrates were evaporated to dryness and dissolved in 1 mL of a water-acetonitrile-methanol mixture (65:17.5:17.5; *v*/*v*/*v*). Extracts were filtered using 0.45 µm porosity disks (Thermo Scientific Fisher, Villebon-Sur-Yvette, France) before analysis. HPLC analysis was performed using a Dionex Ultimate 3000 UHPLC (Thermo Scientific, France) using a 125 × 2 mm, 5 µm, 100 Å, Luna^®^ C18(2) LC column (Phenomenex, Torrance, CA, USA). Aflatoxins were separated using the program described by Fu, Huang, & Min, 2008, with minor modifications [62]. A mixture of water (acidified with 0.2% acetic acid)-acetonitrile (79:21, *v*/*v*) is eluent A and methanol is eluent B. Separation program consists of a 30 min A:B (82.5:17.5) isocratic flow at 0.2 mL/min. Aflatoxins were detected using a fluorescent detector at wavelengths of 365/430 nm (excitation/emission). UV Spectra were confirmed by an additional diode array detector (DAD) coupled to the apparatus. Sample concentrations were calculated based on a standard calibration curve.

### 5.8. Statistics 

All experiments were performed in triplicate. For gene expression assays, six biological replicates were used each time for each gene. Data presented for the dose-dependent effect of hyssop extract were analyzed using a One-way ANOVA followed by a post hoc Dunnett’s test (α = 0.05) and conducted with XLSTAT (Addinsoft, Paris). Gene expression and growth and sporulation data were analyzed using Student’s t-test in Excel statistics. Differences were considered statistically significant when *p*-value was lower than 0.05.

## Figures and Tables

**Table 1:** The effect of the addition of 10 mg/mL hyssop to the culture medium on the development of *A. flavus* (i) colony diameter was measured in length and width; (ii) weight was measured after a 48 h-drying at 60 °C; (iii) germinating conidia were counted by observation under stereo-microscope after 16 h incubation at 27 °C; (iv) total spore count is estimated following a complete wash of conidia and a Malassez-cell count of proper dilutions and (v) spore density was calculated based on the total spore count related to the colony surface. Results are expressed as mean ± SEM (*n* = 3).

Observed Parameters		MEA	MEA + Hyssop 10 mg/mL
**Growth**	Colony diameter (cm)	4.25 ± 0.03	4.4 ± 0.03
	Mycelium dry weight (g)	0.16 ± 0.03	0.15 ± 0.02
**Sporulation**	Germinating conidia after 16 h (%)	96.5 ± 8.5%	101.5 ± 4%
	Total spore count	8.1 × 10^8^ ± 4.5 × 10^7^	1.1 × 10^9^ ± 9.9 × 10^7^
	Spore density (conidia/cm^2^)	5.7 × 10^7^ ± 2.6 × 10^6^	7 × 10^7^ ± 5.6 × 10^6^

## Supplementary Materials

The following are available online at www.mdpi.com/2072-6651/9/3/87/s1, Table S1: Gene expression ratios of AFB_1_ cluster genes upon hyssop addition. Ratios are obtained in comparison to control values.
