# Supplementary material for: Identification of the Anti-Aflatoxinogenic Activity of Micromeria graeca and Elucidation of Its Molecular Mechanism in Aspergillus flavus
Source: Toxins (Basel). 2017 Mar 1;9(3):87. doi: 10.3390/toxins9030087 (PMC5371842; doi:10.3390/toxins9030087)
Supplement: Supplementary file 1 [file toxins-09-00087-s001.pdf]

Supplementary Materials: Identification of the Anti-Aflatoxinogenic Activity of *Micromeria graeca* and Elucidation of its Molecular Mechanism in *Aspergillus flavus*

Rhoda El Khoury, Isaura Caceres, Olivier Puel, Sylviane Bailly, Ali Atoui, Isabelle P. Oswald, André El Khoury and Jean-Denis Bailly

Table S1. Gene expression ratio values of AFB_1 cluster genes upon hyssop addition. Ratios are obtained in comparison to control values.

Gene	Function of encoded protein	Gene expression ratio	SEM	Fold change	*p*-value
AFB₁ cluster genes					
aflR	Zn-finger transcription factor	0.31	0.053	3.2	<0.0001
aflS	AFLR co-activator	0.35	0.069	2.8	<0.0001
aflT	MFS-transporter	0.43	0.055	2.3	<0.0001
aflA	Fatty acid synthase	0.08	0.019	12.2	<0.0001
aflB	Fatty acid synthase	0.08	0.018	12.3	<0.0001
aflC	Polyketide synthase	0.07	0.029	14.7	0.00058
hypC	Noranthrone oxidase	0.01	0.003	167.2	<0.0001
aflD	Ketroreductase	0.06	0.022	16.8	<0.0001
aflE	Deshydrogenase	0.06	0.019	17.8	<0.0001
aflG	Cytochrome P-450 mono-oxygenase	0.11	0.042	9.3	<0.0001
aflH	Deshydrogenase	0.04	0.011	24.8	<0.0001
aflI	Flavin-dependent oxidoreductase	0.02	0.007	60.7	0.00043
aflV	Microsomial Cyt. P-450 mono-oxygenase	0.05	0.012	19.8	<0.0001
aflJ	Esterase	0.06	0.020	18.0	<0.0001
aflK	Cyclase	0.07	0.019	14.9	<0.0001
aflL	Desaturase	0.05	0.015	19.2	<0.0001
aflM	Ketoreductase	0.12	0.053	8.4	0.00120
aflN	Cytochrome P-450 mono-oxygenase	0.03	0.010	34.0	<0.0001
aflX	Oxidoreductase	0.02	0.008	50.4	<0.0001
aflO	O-methyl transferase (I)	0.00	0.000	468.8	<0.0001
aflP	O-methyl transferase (II)	0.03	0.010	29.7	<0.0001
aflQ	Cytochrome P-450 mono-oxygenase	0.07	0.027	13.4	<0.0001
hypB	Hypothetical protein	0.03	0.016	35.8	0.00085
aflW	Cytosolic mono-oxygenase	0.07	0.019	14.7	<0.0001
aflY	Baeyer-Villiger mono-oxygenase	0.02	0.009	45.8	<0.0001
hypD	Integral membrane protein	0.11	0.024	9.4	<0.0001
hypE	Hypothetical protein	0.05	0.011	18.5	0.00018

Table S1. *Cont.*

Gene	Function of encoded protein	Gene expression ratio	SEM	Fold change	*p*-value
Secondary metabolism regulator genes					
abaA	Developmental TF	0.63	0.17	1.58	0.23329
ap-1	bZIP TF (Oxidative stress response)	1.02	0.12	1.02	0.63484
areA	Environmental GATA TF (Nitrogen)	1.71	0.25	1.71	0.0215
atfA	bZIP TF (Oxidative stress response)	0.84	0.14	1.18	0.50247
cat2	Catalase (Oxidative stress response)	0.33	0.04	3.04	<0.0001
catA	Catalase (Oxidative stress response)	0.45	0.05	2.23	0.004
creA	Environmental Zn TF (Carbon)	2.50	0.90	2.50	0.09569
brlA	Developmental Zn finger TF	1.54	0.33	1.54	0.10605
fadA	G-protein signaling pathway	1.47	0.48	1.47	0.78896
fcr3	Global bZIP TF	0.82	0.06	1.22	0.05987
flbA	Developmental regulator	1.40	0.19	1.40	0.07493
fluG	Developmental regulator	0.77	0.12	1.30	0.27419
gprA	G-protein receptor	0.70	0.19	1.44	0.15496
gprG	G-protein receptor	1.33	0.18	1.33	0.09379
gprH	G-protein receptor	0.49	0.01	2.06	0.0006
gprK	G-protein receptor	2.00	0.17	2.00	<0.0001
gprP	G-protein receptor	1.19	0.07	1.19	0.23350
laeA	Velvet complex	1.10	0.18	1.10	0.53537
meaB	Environmental bZIP TF (Nitrogen)	1.14	0.14	1.14	0.31329
mnsod	Mn superoxide dismutase	0.49	0.09	2.04	0.0007
msnA	Zn finger TF (Oxidative stress response)	3.25	0.77	3.25	0.0126
mtfA	Zn finger TF (Oxidative stress response)	1.93	0.17	1.93	0.0001
nsdC	Global Zn finger TF	1.54	0.18	1.54	0.0122
pacC	Environmental Zn finger TF (pH)	1.64	0.11	1.64	<0.0001
ppoA	Oxylipin	0.87	0.05	1.15	0.19087
ppoB	Oxylipn	0.85	0.10	1.18	0.17278
ppoC	Oxylipin	1.50	0.14	1.50	0.003
rasA	Ras-family signaling pathway	2.36	0.65	2.36	0.05524
sod1	CuZn superoxide dismutase	0.60	0.05	1.67	0.013
srrA	bZIP TF (Oxidative stress response)	1.44	0.13	1.44	0.0017
stuA	Developmental factor	1.76	0.44	1.76	0.0012
veA	Velvet complex	3.76	0.44	3.76	<0.0001
velB	Velvet complex	1.03	0.10	1.03	0.63158
vosA	Velvet complex	0.85	0.14	1.05	0.87741
